# The quality of life in women with cervical cancer and precancerous lesions of Han and ethnic minorities in Southwest China

**DOI:** 10.1186/s12885-021-08856-8

**Published:** 2021-10-16

**Authors:** Min Zhao, Xin Pu, Yi Jun Yan, Shao Zhang, Xue Long, Lei Luo, Zheng Li

**Affiliations:** 1 Present Address: Medical Administration Department, The Third Affiliated Hospital of Kunming Medical University (Yunnan Cancer Hospital, Yunnan Cancer Center), 519 Kun Zhou Road, Xi Shan county, Kunming, 650118 Yunnan China; 2 School of Public Health, Kunming Medical University, 1168 Yu Hua Street Chun Rong Road, Cheng Gong New City, Kunming, 650500 Yunnan China; 3 Medical records Statistics Department, The First Affiliated Hospital of Kunming Medical University, 295 Xichang Road, Kunming, 650032 Yunnan China; 4 Department of nuclear medicine, The Affiliated Hospital of Yunnan University, 176 Qingnian Road, Kunming, 650021 Yunnan China; 5 Department of Gynecologic Oncology, The Third Affiliated Hospital of Kunming Medical University (Yunnan Cancer Hospital, Yunnan Cancer Center), 519 Kun Zhou Road, Xi Shan county, Kunming, 650118 Yunnan China; 6 Clinical Laboratory, Kunming Children’s Hospital, 288 Qian Xing Road, Kunming, 650118 Yunnan China

**Keywords:** Cervical cancer, Cervical precancerous lesions, Health-related quality of life, EQ-5D, Cross-sectional study, Ethnic minorities, China

## Abstract

**Background:**

As patients with cervical cancer and precancerous lesions can be diagnosed at early stage and live longer, it is imperative to understand their health-related quality of life so that better cancer-related policies could be promoted and reasonable distribution of limited resources could be implemented. We conducted a cross-sectional study in the Third Affiliated Hospital of Kunming Medical University to assess the health-related quality of life in our targeted population. Due to the characteristics of Yunnan nationality, our study population includes both Han people and ethnic minorities.

**Methods:**

A cross-sectional study was conducted from January 2019 to December 2020, and 300 patients were selected, who were initially diagnosed with cervical cancer and cervical intraepithelial neoplasia (CIN) pathologically. EQ-5D questionnaire was used to evaluate their quality of life.

**Results:**

Patients in Han and ethnic minorities showed good comparability. EQ-5D VAS score was statistically significant between Han and ethnic minorities (mean, 85.42 vs. 81.01; *P*<0.05). EQ-5D utility score was slightly different but without statistical significance between the two groups (mean, 0.959 vs. 0.932; *P*>0.05). Nationality, economic trouble, menopause status and participation of China National Cervical Cancer Screening Program (CNCCSP) are influencing factors of HRQoL among women with cervical cancer and precancerous lesions. Besides, we also found low awareness in the CNCCSP and human papilloma virus vaccine, as well as low participation in the national screening program.

**Conclusion:**

The results of our study imply that the difference of HRQoL does exist between Han people and ethnic minorities with cervical cancer and precancerous lesions. Health providers and health-related departments need to invest more health and financial resources to expand the awareness and participation of the screening project. More efforts should be made in underdeveloped minority areas to assure the accessibility of health resources and interventions. To mitigate economic trouble caused by the diseases, more equal insurance reimbursement should be suggested and implemented in people with or without employee insurance.

## Background

In recent years, tumor has become one of the major diseases that endanger people’s life and health. According to the cancer statistics 2018, cervical cancer ranks fourth for both incidence and mortality among females [1]. Nearly 90% of deaths from cervical cancer each year are of women living in low- and middle- income countries [2]. Cervical cancer refers to a malignant tumor that occurs in the cervical canal, and people aged 40 to 50 are predisposed to cervical cancer. Cervical cancer can be preventable, and it takes years to progress from precancerous lesions to cervical cancer. Persistent infection with specific types of Human papillomavirus viruses (HPV) is a risk factor of cervical cancer, and a diagnosis of precancerous lesions known as cervical intraepithelial neoplasia (CIN) is of great importance in the prevention of cervical cancer. Clinicians mainly focus on clinical treatment and physical disorders of the disease after patients were diagnosed. At present, the treatment of cervical cancer and precancerous lesions includes surgery, chemotherapy and radiotherapy. The above-mentioned procedures paid little attention to the quality of life of the patients, which is exactly the focus of our study.

The incidence and mortality rates of cervical cancer are increasing decades, seriously threatening the health of women in China [3]. With the development of national standardized diagnosis and treatment of cancer prevention and control, more and more cervical precancerous lesions and cancer patients are diagnosed and treated at early stage. Data showed that age-standardized 5-year relative survival rate increased substantially for all cancers over the past decades, and the survival rate of cervical cancer had increased 4.5% (95% CI [2.9–6.2]) [4]. That means cervical cancer survivors or CIN patients diagnosed at early stages have also increased correspondingly and lived longer than before. Given the growing numbers of women with cervical cancer and precancerous lesions, a better understanding of their quality of life resulted from different treatment modalities has become imperative [5]. Health related quality of life (HRQoL) reflects one’s health status because it covers several important health parameters, including physical, psychological, and social health dimensions [6]. HRQoL is able to convey important information for assessing the disease and the effectiveness of interventions as well [7]. Therefore a better understanding of HRQoL in cancer survivors helps raise awareness, promote cancer prevention and control policies, and facilitate better targeted use of limited resources [8]. Evaluation of HRQoL in both cervical cancer and precancerous lesions patients is important in order to design interventions for improving patients’ outcome as well as to monitor and evaluate the effectiveness of treatment and interventions [9].

Health interventions as well as their side effects always influence not only the health status of the patients but also their social and emotional well-being [9]. Some functional disorders occur following therapies such as surgeries and radiotherapy, which adversely impact the quality of life [10]. These therapies involve surgical alteration of female genital anatomy affecting directly their perception of body image and sexual functions; radiotherapy could damage the vaginal mucosa; and chemotherapy causes side effects including nausea, vomiting, diarrhea, constipation, mucositis, weight changes, and hormonal changes [11, 12]. Qualitative research conducted by Ying Chun Zeng et al showed the impact of cervical cancer included physical and psychological sequelae, family distress, financial trouble, and disruptions to their social functioning and sexual life in China [13].

Research of HRQoL has emerged in China in recent decades. It focuses on special populations such as rural population, senior citizens, childbearing-age women, cardiovascular patients, cancer patients and so on. However, few studies have explored the HRQoL following diagnosis and treatment among ethnic minorities. It is believed that cultural values or beliefs owned by the ethnic minorities have impacts on the individuals’ thoughts, interpretations, and behavior [13]. Ethnic minorities are widely distributed in China, but most of them are situated in the western and relatively underdeveloped border areas of China. Yunnan Province with the largest number of ethnic minorities is situated in the southwestern border of China. At the end of 2019, the province’s permanent population was 48.583 million, including ethnic minorities 16.212 million, accounting for 33.6% [14]. In 2015, the incidence and mortality rates of cervical cancer were 9.89 per 100,000 and 3.05 per 100,000 in china [15]. Cervical cancer ranked the fifth malignant tumor in Yunnan province, fourth among females. Its incidence in Yunnan province was 13.61 per 100,000 (age-standardized rate China), and the mortality rate was 3.89 per 100,000 (age-standardized rate China) [16]. In July 2019, the “Healthy China action 2019-2030” launched by the State Council clearly proposed that cervical cancer screening coverage should reach more than 80% by 2030 [17]. However, we also noticed that only 26.7% women had been participated in CNCCSP in China [18], and 21.4% in Yunnan province [19].

This study aims to measure the HRQoL in women with cervical cancer and precancerous lesions both in ethnic minorities and Han people in southwest China, and to compare the differences between the two groups and to explore related factors affecting HRQoL.

## Methods

### Patients

A hospital based cross-sectional study was performed in the Third Affiliated Hospital of Kunming Medical University /Yunnan Cancer Hospital, which is the largest hospital specialized in tumor-related treatment and is also a tertiary hospital in Yunnan province. Patients, who were initially diagnosed as cervical precancerous lesions and cancer pathologically, were selected from the hospital during January 2019 to December 2020. They were interviewed after their treatment within 3 months. Three hundred of patients consented were enrolled for the study, including 2 hundred Han people and 1 hundred ethnic minorities.

The inclusion criteria were patients were: (1) aged 18 years or above, (2) histologically confirmed of cervical cancer or precancerous lesions, (3) previously untreated and newly diagnosed with cervical cancer and precancerous lesion in the hospital, (4) fully confirmed of the diagnosis, (5) identified with a definite clinical stage.

Exclusion criteria were patients had (1) severe co-morbidities such as severe heart or pulmonary diseases, or encephalopathy or those with severe liver or kidney dysfunction, which had major impact on the quality of life (2) other malignant tumors at the same time, (3) contraindications before radiotherapy, chemotherapy and surgery, (4) cognitive dysfunction and were unwilling to take part in the survey.

The stage of cervical cancer was further identified by two gynecologists according to the diagnostic rules of International Federation of Gynecology and Obstetrics (FIGO). Investigators were trained uniformly, and they explained the purpose and the contents to the patients before face-to-face interviews. Informed consents were obtained from all the patients.

### Questionnaire

Europol Five-Dimensional Health Questionnaire (EQ-5D) was used to evaluate the health-related quality of life in this study. EQ-5D is one of the handful of measures recommended for use in cost-effectiveness analyses by the Washington Panel in Health and Medicine [20]. This instrument had been proved with good reliability and validity among cervical cancer patients, also among ethnic minority (Uygur) in China [21, 22]. EQ-5D is more responsive than most of the condition-specific measures, and is used increasingly in 1) monitoring the health status of patient groups 2) assessing the seriousness of conditions at different moment in time; 3) providing evidence about medical effectiveness; 4) economic studies; and 5) establishing levels of population health status both locally and nationally [20].

EQ-5D is composed of two parts including a descriptive system with five dimensions and a visual analogue scale (VAS). The five dimensions refer to mobility, self-care, usual activities, pain and discomfort, and anxiety and depression. The VAS score is a subjective evaluation of health status by the patients themselves, and the score ranges between 100 (the best imaginable health) and 0 (the worst imaginable health) [23]. This survey adopted the superior version of EQ-5D-5L, with each dimension divided into five levels: 1) no problem 2) slight 3) moderate 4) severe 5) extreme. The EQ-5D-5L is accurate in evaluating the changes of health-related quality of life. Given that the utility score of EQ-5D health status may be affected by culture [24], Chinese value sets [25] were used to produce utility score in this study. Respondents were also asked to rate on visual analogue scale (VAS).

In addition, the questionnaire also included sociodemographic and clinic characteristics. Sociodemographic information included age, educational level, marital status, number of pregnancies/ children, and annual household income. Moreover, this survey also studied the awareness and participation of the CNCCSP, as well as the awareness of human papilloma virus (HPV) vaccine. Medical characteristics involved mode of case-finding, menopausal status, pathological classification and treatment, degree of pathological differentiation, contraceptive method, cervical precancerous lesions, and cancer stages (FIGO). All the variables above were acquired both from self-reported and medical records review.

### Statistical analysis

EpiData 3.1(EpiData Entry 3.1.2071.2008) was adopted for data input and SPSS 23.0 (IBM Corp, Armonk, NY, USA) for data analysis. Number and percentage were used to describe variables in sociodemographic and clinic characteristics. Means and standard deviations were used to describe the EQ-5D utility score and VAS score. A t-test and one way analysis of variance (ANOVA) were conducted to compare the differences between ethnic minorities and Han people for continuous variables, and LSD-T (least significant difference t test) was then further conducted for pairwise comparison if overall difference was statistically different. The chi-square test was used to compare the proportion of each component. Statistical significance was based on two-tailed *P*-values, and p<0.05 was considered statistically significant.

Linear regression analysis method was used to explore predictors variables, and the dependent variable was EQ-5D score, including both VAS score and utility score; while the independent variables were age group, marital status, annual household income, menopause status, participation of the screening, employee insurance, economic trouble, treatment and cancer stage. The inclusion criterion α was ≤0.05, and the exclusion criterion α was ≥0.10. Inspection level α = 0.05.

## Results

### Characteristics

Table 1 showed the social demographic characteristics of the 300 cervical cancer patients, among which one third was ethnic minorities and two thirds Han people. The statistical analysis showed good comparability between the two groups in social demographic characteristics.

**Table 1:** Social demographic of study population

	Han people	Ethnic minorities	Total	*P*
Age in years				
20–29 years	8 (4.0%)	5 (5.0%)	13 (4.3%)	0.987
30–39 years	40 (20.0%)	21 (21.0%)	61 (20.3%)	
40–49 years	87 (43.5%)	41 (41.0%)	128 (42.5%)	
50–59 years	54 (27.0%)	28 (28.0%)	82 (27.3%)	
60–69 years	11 (5.5%)	5 (5.0%)	16 (5.3%)	
Educational Status (years of education)				
Illiteracy (0)	31 (15.5%)	23 (23.0%)	54 (18.0%)	0.065
Primary (1–9)	125 (62.5%)	57 (57.0%)	182 (60.7%)	
Middle (10–12)	24 (12.0%)	5 (5.0%)	29 (9.7%)	
College or above (≥13)	20 (10.0%)	15 (15.0%)	35 (11.7%)	
Marital status				
Married	186 (93.0%)	92 (92.0%)	278 (92.7%)	0.754
Others	14 (7.0%)	8 (8.0%)	22 (7.3%)	
Number of pregnancies				
No pregnancy	2 (1.0%)	2 (2.0%)	4 (1.3%)	0.703
≤ 3	113 (56.5%)	59 (59.0%)	172 (57.3%)	
4–7	83 (41.5%)	37 (37.0%)	120 (40.0%)	
≥ 8	2 (1.0%)	2 (2.0%)	4 (1.3%)	
Number of Children				
NO	5 (2.5%)	5 (5.0%)	10 (3.3%)	0.521
≤ 2	157 (78.5%)	76 (76.0%)	233 (77.7%)	
≥ 3	38 (19.0%)	19 (19.0%)	57 (19.0%)	
Annual Household Income (RMB)				
≤ 2499	69 (34.5%)	33 (33.0%)	102 (34.0%)	0.983
2500–4999	58 (29.0%)	27 (27.0%)	85 (28.3%)	
5000–7499	28 (14.0%)	15 (15.0%)	43 (14.3%)	
7500–9999	10 (5.0%)	6 (6.0%)	16 (5.3%)	
≥ 10,000	35 (17.5%)	19 (19.0%)	54 (18.0%)	
Having Employee insurance or not				
Yes	34 (17.0%)	20 (20.0%)	54 (18.0%)	0.524
No	166 (83.0%)	80 (80.0%)	246 (82.0%)	
Economic trouble				
No	32 (16.0%)	19 (19.0%)	51 (17.0%)	0.796
Moderate	60 (30.0%)	28 (28.0%)	88 (29.3%)	
Extreme	108 (54.0%)	53 (53.0%)	161 (53.7%)	
Awareness of China National Cervical Cancer Screening Program (CNCCSP)				
Yes	76 (38.0%)	35 (35.0%)	111 (37.0%)	0.612
No	124 (62.0%)	65 (65.0%)	189 (63.0%)	
Participation of China National Cervical Cancer Screening Program (CNCCSP)				
Yes	49 (24.5%)	21 (21.0%)	70 (23.3%)	0.499
No	151 (75.5%)	79 (79.0%)	230 (76.7%)	
Awareness of human papilloma virus (HPV) vaccine				
Yes	76 (38.0%)	33 (33.0%)	109 (36.3%)	0.369
No	124 (62.0%)	67 (67.0%)	191 (63.7%)	

90% of the patients of both groups were between 30 and 59 years old, and the proportions of different age groups were similar between Han people and ethnic minorities.

In terms of education, overall, 18.0% of patients were illiterates, and 60.7% of them had only primary education; about 10% of the patients had finished high school education and 11.7% of them accepted university education. Although the proportions of educational levels were slightly different between the two groups, the data showed no statistical difference.

Most of the patients were married, the proportions of the married in Han people and ethnic minorities were 93.0 and 92.0%.

About 60.0% of patients had less than three pregnancy histories in both groups. 41.5% of Han and 37.0% of ethnic minorities had four to seven pregnancies. The proportions of women without children were 2.5 and 5.0% in Han and ethnic minorities. The majority (78.5% in Han, 76.0% in ethnic minorities) of patients had one or two children. The proportions of women with three or more children were both 19.0% in the two groups.

The social demographic comparisons between Han people and ethnic minorities showed no statistical significance in annual household income, employee insurance status, and economic trouble. By self-report, the proportion of moderate and heavy economic trouble caused by cervical cancer in Han people was 84.0%, while 81.0% in ethnic minorities; the proportion of those who reported did not have economic trouble with cervical cancer and precancerous lesions were 16.0 and 19.0% in Han and ethnic minorities, which also showed no difference between the two groups.

The study also investigated the awareness and participation of the CNCCSP as well as the awareness of HPV vaccine among the patients. The proportions of being aware of the screening program were 38.0 and 35.0% in Han and ethnic minorities. The proportions of participation in the CNCCSP were lower than those of the awareness, 24.5 and 21.0% in Han and ethnic minorities. The proportions of being aware of HPV vaccine and the CNCCSP were almost the same.

In China, compulsory education lasts 9 years, including primary and secondary school, and the educational levels of the two groups were very similar.

Clinic characteristics including mode of case-finding, menopause status, pathological classification, treatment, degree of tumor differentiation, contraception, and cancer stage were also compared between Han people and ethnic minorities. The results showed good homogeneity and comparability between the two groups (Table 2).

**Table 2:** Clinical characteristics of study population

	Han people	Ethnic Minorities	Total	*P*
Mode of case-finding				
Clinical symptoms	126 (63.0%)	58 (58.0%)	184 (61.3%)	0.420
Health examination	36 (18.0%)	22 (22.0%)	58 (19.3%)	
Screening	38 (19.0%)	19 (19.0%)	57 (19.0%)	
Others	0 (0.0%)	1 (1.0%)	1 (0.3%)	
Menopause status				
Yes	69 (34.5%)	34 (34.0%)	103 (34.3%)	0.931
No	131 (65.5%)	66 (66.0%)	197 (65.7%)	
Pathological classification				
Squamous cell neoplasms	99 (49.5%)	47 (47%)	146 (48.7%)	0.908
Adenocarcinoma	18 (9.0%)	10 (10%)	28 (9.3%)	
Others	83 (41.5%)	43 (43%)	126 (42.0%)	
Treatment				
Cervical Conization	63 (31.5%)	34 (34.0%)	97 (32.3%)	0.523
Radical Hysterectomy	77 (38.5%)	30 (30.0%)	107 (35.7%)	
Chemoradiotherapy	46 (23.0%)	28 (28.0%)	74 (24.7%)	
Adjuvant therapy	14 (7.0%)	8 (8.0%)	22 (7.3%)	
Degree of tumor differentiation				
Not stated/given	88 (44.0%)	47 (47.0%)	135 (45.0%)	0.400
Well-differentiated	12 (6.0%)	3 (3.0%)	15 (5.0%)	
Moderately differentiated	47 (23.5%)	29 (29.0%)	76 (25.3%)	
Poorly differentiated	53 (26.5%)	21 (21.0%)	74 (24.7%)	
Contraceptive methods				
IUD	123 (61.5%)	63 (63.5%)	186 (62.0%)	0.699
Condom	35 (17.5)	14 (14.0%)	49 (16.3%)	
Other	23 (11.5%)	10 (10.0%)	33 (11.0%)	
No	19 (9.5)	13 (13.0%)	32 (10.7%)	
Stages				
CIN	72 (36.0%)	38 (38.0%)	110 (36.7%)	0.960
Stage I	64 (32.0%)	28 (28.0%)	92 (30.7%)	
Stage II	36 (18.0%)	18 (18.0%)	54 (18.0%)	
Stage III	24 (12.0%)	14 (14.0%)	38 (12.7%)	
Stage IV	4 (2.0%)	2 (2%)	6 (2.0%)	

Most cervical cancer and precancerous lesions cases were detected through abnormal clinical symptoms such as abnormal vaginal bleeding or abnormal leucorrhea, and the proportions of this case-finding were 63.0 and 58.0% in Han people and ethnic minorities. Health examinations and screening were other important ways of case-finding, and the proportions in both groups were about 40%. About 34% of study patients in both groups were menopausal. Squamous cell neoplasms were the most found in the study population, accounting for almost half in both Han and ethnic minorities. 31.5% of Han and 34.0% of ethnic minorities underwent cervical conization. The proportions of radical hysterectomy, chemoradiotherapy, and adjuvant therapy in Han people were 38.5, 23.0 and 7.0%; and 30.0, 28.0 and 8.0% in ethnic minorities.

Among patients with available data on cancer stage, 36.0% presented with CIN, 32.0% with stage I, 18.0% with stage II, 12.0% with stage III and 2.0% with stage IV in Han people; they were 38.0, 28.0, 18.0 14.0 and 2.0% in the ethnic minorities.

Moreover, the data also showed no statistically significant difference in the degree of pathological differentiation and contraceptive methods between the comparison of the two groups.

To sum up, no significant difference existed between Han and ethnic minorities in terms of sociodemographic characteristics and clinical characteristics. Both Han and ethnic minorities had very good comparability.

### Comparison of EQ-5D

Generally, the VAS score of Han (85.42, SD = 13.54) was higher than that of the ethnic minorities (81.01, SD = 15.54) with statistically difference (*p* = 0.012), and the EQ-5D utility scores in Han and ethnic minorities were (0.959, SD = 0.075, 0.932, SD = 0.159) without statistically difference (*P* = 0.108).

The comparison of EQ-5D-5L, both VAS and utility scores, was conducted in Han and ethnic minorities separately in terms of different age groups, marital status, annual household income, HPV vaccine awareness, employee insurance status, economic trouble, menopause status, pathological classification, different treatments, and stages. See Table 3.

**Table 3:** The comparisons of Health-related Quality of life in different variables based on EQ-5D

	Number		VAS score, Mean (SD)		EQ-5D utility score Mean (SD)	
	Han	Ethnic	Han	Ethnic	Han	Ethnic
Age in Years						
≤ 39	48	26	83.42 (13.65)	79.73 (13.10)	0.974 (0.048)	0.938 (0.164)
40–59	141	69	85.48 (13.73)	81.71 (16.72)	0.952 (0.083)	0.930 (0.163)
≥ 60	11	5	93.27 (6.40)	78.00 (10.95)	0.985 (0.034)	0.939 (0.077)
*P* value			0.092	0.781	0.108	0.971
Marital status						
Married	186	92	85.67 (13.53)	80.72 (15.84)	0.959 (0.074)	0.932 (0.165)
Others	14	8	82.00 (13.61)	84.38 (11.78)	0.947 (0.077)	0.941 (0.082)
*P* value			0.329	0.526	0.555	0.876
Annual Household Income (RMB)						
≤ 2499	69	33	84.09 (16.30)	79.24 (17.85)	0.953 (0.095)	0.941 (0.143)
2500–4999	58	27	85.41 (12.11)	80.00 (15.87)	0.965 (0.064)	0.893 (0.242)
5000–7499	28	15	86.39 (13.32)	83.20 (15.28)	0.958 (0.052)	0.956 (0.080)
7500–9999	10	6	83.50 (15.28)	85.00 (10.49)	0.930 (0.096)	0.869 (0.150)
≥ 10,000	35	19	87.80 (9.03)	82.53 (12.92)	0.973 (0.053)	0.974 (0.034)
*P* value			0.722	0.847	0.500	0.366
Participation of China National Cervical Cancer Screening Program (CNCCSP)						
Yes	49	21	86.53 (12.37)	85.95 (7.85)	0.976 (0.047)	0.982 (0.026)
No	151	79	85.05 (13.91)	79.70 (16.80)	0.954 (0.081)	0.919 (0.177)
*P* value			0.058	0.017	0.020	0.003
Awareness of human papillomaviruses vaccine						
Yes	76	33	83.76 (14.67)	83.24 (11.37)	0.971 (0.057)	0.971 (0.052)
No	124	67	86.43 (12.75)	79.91 (17.20)	0.952 (0.084)	0.913 (0.189)
*P* value			0.177	0.251	0.082	0.023
having an employee insurance statue or Not						
Yes	34	20	89.21 (9.36)	82.75 (13.03)	0.968 (0.058)	0.977 (0.026)
No	166	80	84.64 (14.14)	80.58 (16.15)	0.958 (0.078)	0.921 (0.176)
*P* value			0.022	0.578	0.369	0.007
Economic Trouble						
No	32	19	88.34 (9.22)	84.53 (10.56)	0.986 (0.039)	0.962 (0.072)
Moderate	60	28	84.63 (13.91)	82.46 (15.32)	0.955 (0.070)	0.963 (0.073)
Extreme	108	53	84.98 (14.37)	78.98 (17.00)	0.954 (0.084)	0.906 (0.205)
*P* value			0.407	0.350	0.094	0.209
Menopause status						
Yes	69	34	88.55 (11.31)	82.29 (15.66)	0.956 (0.090)	0.902 (0.185)
No	131	66	83.76 (14.34)	80.35 (15.55)	0.961 (0.066)	0.948 (0.143)
*P* value			0.011	0.556	0.601	0.174
Treatment						
Cervical Conization	63	34	87.83 (11.45)	81.65 (13.71)	0.971 (0.056)	0.974 (0.413)
Radical Hysterectomy	77	30	83.78 (15.29)	75.90 (16.80)	0.954 (0.070)	0.875 (0.260)
Chemoradiotherapy	46	28	85.43 (12.87)	86.89 (13.57)	0.959 (0.061)	0.939 (0.105)
Adjuvant therapy	14	8	83.50 (13.84)	76.88 (19.44)	0.940 (0.168)	0.945 (0.065)
P value			0.336	0.046	0.408	0.093
Stage						
CNI	72	38	86.58 (13.96))	82.00 (13.30)	0.970 (0.059)	0.968 (0.050)
Stage I	64	28	86.00 (12.23)	76.46 (17.02)	0.954 (0.068)	0.870 (0.268)
Stage II	36	18	81.06 (15.05)	79.11 (18.34)	0.938 (0.116)	0.953 (0.081)
Stage III	24	14	86.75 (13.44)	88.57 (12.92)	0.977 (0.040)	0.929 (0.126)
Stage IV	4	2	86.25 (9.47)	90.00 (0.000)	0.936 (0.098)	0.951 (0.000)
P value			0.328	0.145	0.157	0.160
CIN or cancer						
CIN	72	38	86.58 (13.96)	82.00 (13.30)	0.970 (0.059)	0.968 (0.050)
Cervical cancer	128	62	84.76 (13.30)	80.40 (16.84)	0.953 (0.082)	0.910 (0.196)
P value			0.361	0.620	0.123	0.029

### VAS score comparison

In the VAS score comparison of Han people, statistical differences were found in employee insurance status and menopause status. Han patients with an employee insurance had higher VAS scores, 89.21(SD = 9.36), than those without, 84.64(SD = 14.14), with *P* value 0.022. The VAS scores of menopausal women were higher than those of the non-menopausal women, 88.55 (SD = 11.31)) and 83.76 (SD = 14.34) (*P* = 0.011).

Ethnic minorities who participated in the screening program had higher VAS scores, 85.95 (SD = 7.85) compared with those who did not, 79.70 (SD = 16.80), and the difference was statistically significant (*P* = 0.017); Comparisons of the VAS scores of different treatments in the ethnic minorities showed significant statistical differences (*P* = 0.046). Ethnic minorities who underwent chemoradiotherapy had the highest VAS score, 86.89 (SD = 13.57), and those who underwent radical hysterectomy had the lowest VAS score, 75.90 (SD = 16.80).

### EQ-5D utility score comparison

In EQ-5D utility score comparison, the scores of Han and ethnic minorities who participated in the CNCCSP were higher than those who did not. Han patients who participated the CNCCSP got 0.976 (SD = 0.047) and 0.954 (SD = 0.081) for those who didn’t, with *P* value = 0.020; the ethnic minorities who participated the CNCCSP got 0.982 (SD = 0.026) and 0.919 (SD = 0.177) for those who didn’t, with *P* value = 0.003.

Statistical differences were also found in the awareness of HPV vaccine, employee insurance status, and CIN/cancer among subgroups of ethnic minorities. The results in Table 4 showed that ethnic minorities patients being aware of HPV vaccine got higher utility score (0.971, SD = 0.052) than those not (0.913, SD = 0.189) with *P* value = 0.023.; The EQ-5D utility score of the ethnic minorities with an employee insurance was 0.977 (SD = 0.026), and those without was 0.921 (SD = 0.176), with *P* value = 0.007; The EQ-5D utility score of the ethnic minorities with CIN was 0.968 (SD = 0.050), and the score with cervical cancer was 0.910 (SD = 0.196) with *P* value = 0.029.

**Table 4:** Determinants of health-related quality of life (VAS score and utility score) among cervical precancerous lesions and cancer

Dependent variable	Variable	Beta	*P*-value	95%CI
VAS score	Nationality (Han/Ethnic)	−0.147	0.010	−7.864 to −1.084
	Economic trouble	−0.120	0.037	−4.419 to −0.140
	Menopause status	−0.147	0.011	−7.833 to −1.017
EQ-5D utility score	Nationality (Han/Ethnic)	−0.114	0.046	−0.053 to −0.001
	Economic trouble	−0.129	0.024	−0.035 to − 0.003
	Participation of screening	−0.127	0.026	−0.062 to − 0.004

No statistical differences were found in either the EQ-5D utility score or comparisons among subgroups of Han patients including age group, marital status, annual household income, participation of national screening, awareness of HPV vaccine, employee insurance status, economic trouble, menopause status, different treatments and stages.

The results of multiple linear regression implied that the influencing factors of the HRQoL were nationality, economic trouble, menopause status and participation of CNCCSP. The outcomes of linear regression were shown in Table 4.

## Discussion

Evaluating and addressing health-related quality of life issues is an important part of the whole package of modern medical care [26]. Advanced cervical cancer and its related death are internationally regarded as indicators of medical accessibility and health equity [11]. It is particularly important to use appropriate measurement tools to evaluate and improve the HRQoL of these patients, and to evaluate the effectiveness of treatment interventions [27].

Unlike other studies, this study included both Han and ethnic minorities patients. This study provides us with a different perspective to understand health-related quality of life among targeted populations.

The age being diagnosed in this study was consistent with Wanghe’s research, which revealed that the incidence of cervical cancer increased at age thirty to forty, and reached the peak in the age group of 44–49 [28]. Moreover, the comparisons of sociodemographic and clinic characteristics among Han and ethnic minorities demonstrated good comparability (Table 1 and Table 2).

Generally, the VAS score of the ethnic minorities in this study (81.01, SD = 15.54) was consistent with the results of Sang Shuping’s study (81.21, SD = 13.68), conducted specially in Yunnan province in 2013 [29]; the VAS score of Han people was higher in our study (85.42, SD = 13.54) compared with Sang Shuping’s study (80.84). EQ-5D utility score of Han (0.959, SD = 0.075) in our study was also close to Sang’ study (0.952) [29], but the utility score of the ethnics was lower (0.932, SD = 0.159). Moreover, the EQ-5D utility score of patients with precancerous lesions was very close to a study in China [30], whose utility score of precancerous lesions was 0.98 (SD = 0.54) exactly 3 months after treatment; The utility scores were 0.970 (SD = 0.059) and 0.968 (SD = 0.050) in Han people and the ethnic minorities in this study. At the same time, we also noticed our EQ-5D utility score was much higher compared with similar studies in Indonesian, India, and Ethiopia [10, 31, 32]; the VAS score of Han people was close to MA Li’s et al. research [33] (86.23, SD = 11.13), while the utility scores of both Han and the ethnic minorities were higher than those of MA’s research [33] (0.917, SD = 0.013). The big differences of VAS and utility scores compared with other countries’ studies are related to the different value sets used, which can be explained by methodological differences used in the valuation studies, as well as the cultural dissimilarities across the countries [32]. As mentioned before, we used the Chinese EQ-5D-5L value set to calculate the utility score in this study. The utility score based on Chinese value set ranges from − 0.391 to 1.000 [25]. As a whole, the HRQoL of both Han and the ethnic minorities were similar to the general populations in the follow-up studies [34] and in other countries [35].

The comparisons of VAS and utility scores of different sociodemographic variables did not show statistical differences among the subgroups of research population in my study, including age, marital status, annual household income and so on. That might be associated with the small sample sizes of the subgroups (Table 3). Both VAS and utility scores of married patients were higher than those with other marital status in Han people. It was postulated that married patients got more mental and social supports, although the results did not show statistical difference; the opposite results may be related to the small sample size in the ethnic minorities, with only 8 patients of other marital status. In addition, the VAS and utility scores of patients with the highest annual income were higher than those with low income, although there was no statistical significance. Similarly, in terms of economic trouble caused by the diseases, the worse the economic trouble was, the lower the quality of life was, still without statistically significant difference.

Our results confirmed the conclusion made by Chen Hongda et al. on the CNCCSP: there are still some problems in the CNCCSP, such as poor awareness of prevention, low participation rate of screening, lack of individualized and accurate screening programs [36]. In developed countries, the incidence of cervical cancer has dropped significantly, largely due to the early diagnosis and treatment of cervical precancerous lesions [37].

Cervical cancer screening based on cytology has been promoted in some pilot counties in China since 2009. However, insufficient coverage of both the areas and the populations involved led to low chances of screening, so the overall screening rate is still low [38]. A national survey in 2010 showed that only 21% of women have had a Pap smear [39]. As we can see from Table 1, the proportions of being aware of CNCCSP and HPV vaccine were less than 40% in Han and the ethnic minorities; the proportion of participation of the screening was much lower, and they were 24.5 and 21.0% in Han and the ethnic minorities. Nevertheless, the VAS and utility score of patients who had participated in the CNCCSP were higher than those who didn’t in both Han and the ethnic minorities. This suggested that the patients who proactively participated in the CNCCSP had good health awareness and good health status. The results in Table 4 also implied that the participation of the screening was a statistical predictor of EQ-5D utility score. The research population who had participated the CNCCSP were prone to got higher utility score.

It has been proved that HPV vaccination can effectively prevent related precancerous lesions and persistent infection [11]. Since 2016, the World Health Organization has recommended that countries should include the HPV vaccine in their national immunization programs. The HPV vaccine is available in China, but the targeted population who want to be vaccinated should pay the cost (three doses cost 1740–3894 RMB, 269–602 USD) at their own expense, which is relatively high for them. In our study, only 33.0% ethnic minorities knew HPV vaccine, and 38.0% in Han people (Table 1). Therefore, it is worthwhile for the health providers to expand the screening program coverage in ethnic enclave, so that more ethnic women can benefit from CNCCSP.

The reason why patients with an employee insurance got higher VAS and utility scores is due to the advantages of the employee insurance system in China: the reimbursement of hospitalization for the employees is higher. An employee insurance covers the majority of the costs for follow-up treatment of cancer [40, 41].

Different treatments lead to different outcomes of HRQoL. Patients with precancerous lesions tended to undergo conization, which showed the highest VAS and utility scores among all treatments. However, the difference resulted from different treatments was not obvious in this study, which was consistent with studies comparing HRQoL of patients with different treatments [5, 34, 35]. Surprisingly, different from other studies [9, 10, 33, 42], our results did not show a downward trend of VAS and utility scores with the increase of tumor stage. That might be related to the small sample sizes of patients at different stages.

The results of multiple linear regression implied four influencing factors in our study, and they are nationality, economic trouble, menopause status and participation of CNCCSP. Compared with Han patients, HRQoL of ethnic minorities was lower. Nationality, which was a statistically significant predictor for both VAS score and utility score, possibly implied that personal or cultural beliefs of different ethnics had influences on their attitudes towards health. Economic trouble directly affected the overall quality of the patients’ life. The heavier the economic trouble, the worse the quality of life. Patients participated in the screening seemingly had stronger awareness of health status, therefore they had higher evaluation of health-related quality of life than those who did not. Lower utility score in menopausal women could be related to hormonal change and following physiological and psychological changes. However, the comparison of VAS score showed different results. This may require further research.

## Conclusion

The results of our study implied that differences of health-related quality of life exist between Han people and the ethnic minorities in women with cervical cancer and precancerous lesions. The awareness of CNCCSP and HPV vaccine is low among women with cervical cancer and precancerous lesions in the southwest of China. Most women had not even been screened. Nationality, economic trouble, menopause status and participation of screening can influence HRQoL among women with cervical cancer and precancerous lesions. It is necessary for the health providers and health-related departments to invest more resources including health and financial resources to expand the awareness and participation of national screening projects.

It could be helpful to strengthen the awareness of CNCCSP and HPV vaccine so that the targeted population can pay more attention to health. Patients can benefit from disease prevention and early-diagnosis and get higher HRQoL. More efforts should be made in the underdeveloped minority areas to assure the accessibility of health resources. Also interventions designed specifically for ethnic minorities are needed. More equal insurance reimbursement should be suggested and implemented in people with or without employee insurance, so that economic trouble caused by the diseases could be mitigated.

### Limitation

This study has some limitations. Firstly, the sample size is small, especially the patients in stage IV. Secondly, the cross-sectional study design employed means causal conclusions could be made with caution. Thirdly, other factors such as the prevalence of chronic diseases and the quality of sexual life were not considered. Finally, blank control is absent in this study.

## Data Availability

Data are available upon reasonable request from the corresponding author. Email: lengyueds@outlook.com

## Abbreviations

CIN: Cervical intraepithelial neoplasia
HRQoL: Health-related quality of life
HPV: Human papillomavirus viruses
CI: confidence interval
VAS: Visual Analog Scale
LSD-T: least significant difference t test
CNCCSP: China National Cervical Cancer Screening Program (CNCCSP)
